# Synthesis and Characterization of Pure Copper Nanostructures Using Wood Inherent Architecture as a Natural Template

**DOI:** 10.1186/s11671-018-2543-0

**Published:** 2018-04-24

**Authors:** Youming Dong, Kaili Wang, Yi Tan, Qingchun Wang, Jianzhang Li, Hughes Mark, Shifeng Zhang

**Affiliations:** 1 MOE Key Laboratory of Wooden Material Science and Application, Beijing Forestry University, Beijing, 100083 China; 2 Wood Material Technology, Department of Bioproducts and Biosystems, School of Chemical Engineering, Aalto University, 02150 Espoo, Finland; 3 School of Technology, Beijing Forestry University, Beijing, 100083 China

**Keywords:** Copper nanoparticles, Wood template, Hierarchical structure, Stability

## Abstract

The inherent sophisticated structure of wood inspires researchers to use it as a natural template for synthesizing functional nanoparticles. In this study, pure copper nanoparticles were synthesized using poplar wood as a natural inexpensive and renewable template. The crystal structure and morphologies of the copper nanoparticles were characterized by X-ray diffraction and field emission scanning electron microscopy. The optical properties, antibacterial properties, and stability of the hybrid wood materials were also tested. Due to the hierarchical and anisotropic structure and electron-rich components of wood, pure copper nanoparticles with high stability were synthesized with *fcc* structure and uniform sizes and then assembled into corncob-like copper deposits along the wood cell lumina. The products of nanoparticles depended strongly on the initial OH^−^ concentration. With an increase in OH^−^ concentration, Cu_2_O gradually decreased and Cu remained. Due to the restrictions inherent in wood structure, the derived Cu nanoparticles showed similar grain size in spite of increased Cu^2+^ concentration. This combination of Cu nanostructures and wood exhibited remarkable optical and antibacterial properties.

## Background

Metal nanoparticles have garnered wide attention in the scientific community thanks to their exceptional physical and chemical properties [1]. Silver and gold have attracted particularly great interest given their unique plasmon resonance and high stability. However, the high cost of silver and gold limits their wide industrial application [2]. Because copper is much cheaper and more abundant, copper nanoparticles (Cu NPs) may be considered a replacement for silver and gold NPs. Moreover, Cu-based NPs are gaining importance thanks to their catalytic, optical, antibacterial, and electrical conducting properties [3–5]. To fully utilize these properties, the size, purity, and shapes of copper must be well controlled. Therefore, various attempts have been proposed to synthesize NPs with a controlled shape and a specific size distribution, such as solution reduction, thermal decomposition, metal vapor synthesis, radiation methods, microemulsion techniques, mechanical attrition, and electrodeposition [6–10]. Among these, the solution reduction approach is a feasible and exceptionally versatile method for the preparation of Cu NPs. However, it is common to find nanoparticle molecules with spherical shapes; controlled NPs synthesis with other distinct surface morphologies can be accomplished using some unique organic/inorganic templates [11]. Nevertheless, the template consumption in the preparation process is costly, and the procedure is tedious [12].

Another issue in utilizing these Cu NPs is their inherent propensity for surface oxidation in air and resultant aggregation [13]. To avoid this problem, an inert environment (e.g., nitrogen or argon) is used [14]. Other reports have presented various approaches that attempt to address the oxidation problem; such methods are generally based on minimizing exposure of the Cu NPs to oxygen through a protective layer at the particle surface. This layer may consist of polymers [15], organic ligands [16, 17], carbon and graphene [18], or inert metal [19]; however, these strategies require complex processes and/or special equipment.

**Fig. 1:**
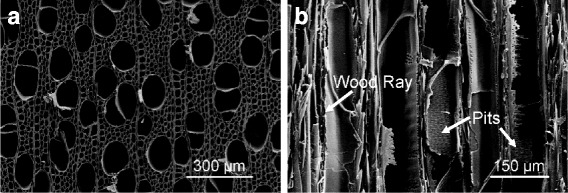
Microstructure of poplar wood. **a** Cross section. **b** Longitudinal section

Wood can be considered a natural template due to its sophisticated structure. As shown in Fig. 1, wood possesses a porous structure from nanoscale to microscale, which provides accessibility to introduce functional materials. Keplinger et al. used wood structure as a mechanically stable scaffold for stimuli responsive gels [20]. Our previous study indicated that wood can be used as the template for assembled ZnO nanostructures [21]. Hybrid wood materials exhibit extraordinary performance in thermal stability, ultraviolet resistance, and antibacterial properties. Due to the inherent hierarchical and anisotropic structure of wood, NP growth within the wood structure is likely to form a 3D order that presents facetted shapes [22]. For instance, magnetic wood with anisotropy can be prepared via co-precipitation of ferric and ferrous ions, and the layered nanosize particles can attach firmly to the inner wood cell wall surface [23]. Therefore, wood is an ideal template to combine with NPs to produce inexpensive, lightweight, and multifunctional materials.

In addition to the unique structure of wood, its lignocellulosic nature—composed of cellulose, lignin, and hemicelluloses—has a reducing and stabilizing effect on metal NPs given the electron-rich features of hydroxyl and phenolic groups in these components [24]. Lin [25, 26] demonstrated that Pt NPs and Ag NPs with a controlled size and shape were successfully synthesized using wood nanomaterials in aqueous systems without employing any other reductants. They attributed the formation of NPs to the reducibility of hydroxyl and phenolic groups in wood components that reduce Pt ions and Ag ions. However, the sophisticated structure of wood has been underused such that the generated Cu NPs have been susceptible to oxidation in previous studies. Hence, wood components appear to be beneficial to NP stability if the NPs are synthesized using solid wood as a template.

In this study, we reported the success of a novel Cu architecture via chemical reduction within poplar wood as the natural template. The morphologies and crystal structure of the Cu NPs were characterized, and the stability, optical properties, and antibacterial properties of the hybrid wood materials were investigated.

## Methods/Experimental

### Materials

From the sound sapwood portions of poplar (*Populus tomentosa* Carr.), samples with a dimension of 50 × 50 × 5 (longitudinal) mm^3^ were prepared and oven-dried at 103 °C to a constant weight.

Copper (II) chloride dehydrate (CuCl_2_·2H_2_O) and sodium borohydride (NaBH_4_) were purchased from Sinopharm Chemical Reagent Co., Ltd. (Shanghai, China). Other analytical-grade chemical reactants were obtained from Beijing Chemical Reagents Co., Ltd. (Beijing, China).

### Preparation of Wood/Cu Composites

**Fig. 2:**
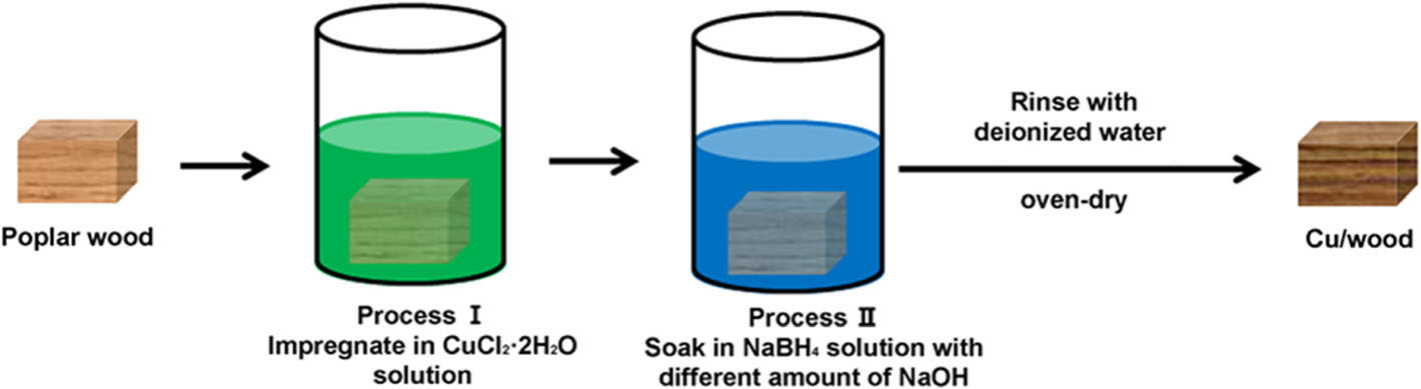
Fabrication process of hybrid wood materials

The fabrication process of hybrid wood materials is shown in Fig. 2. NaBH_4_ was used as the reducing agent for CuCl_2_•2H_2_O. The concentration of the NaBH_4_ and CuCl_2_•2H_2_O solutions was prepared by adding stoichiometric NaOH. Wood samples were dipped in the CuCl_2_•2H_2_O solution under a vacuum (ca. 0.095 MPa) for 30 min and were soaked under atmospheric pressure for 2 h for in-depth diffusion into the porous wood structure. After impregnation, the samples were rapidly immersed in 200 mL NaBH_4_ solution with different amounts of NaOH for 48 h. The samples were then rinsed with deionized water until the pH value was neutral before being oven-dried at 30 °C for 72 h. Detailed formulations of these solutions are listed in Table 1.

**Table 1:** Solution formulations

Groups	CuCl_2_ (mol/L)	NaBH_4_ (mol/L)	NaOH (mol/L)
A	0.5	0.25	0.5
B	0.5	0.25	0.8
C	0.5	0.25	1.0
D	0.5	0.25	1.2
E	1.0	0.50	1.0
F	1.5	0.75	1.0

### Characterization of Cu Nanostructures

The X-ray diffraction (XRD) measurements of the NPs were carried out using a Bruker D8 advance diffractometer (Germany). The apparatus parameters were set as follows: Cu-Kα radiation with a graphite monochromator, voltage 40 kV, electric current 40 mA, and 2θ scan range from 5° to 90° with a scanning speed of 2°/min.

The morphologies of Cu nanostructures were examined using a field emission scanning electron microscope (FE-SEM, Hitachi SU8010, Japan) equipped with an energy dispersive X-ray spectroscope (EDS, EX-350, Horiba Scientific, Japan). The interior portions of longitudinal planes in the sample were mounted on conductive adhesives and were coated with gold sputter followed by observation using FE-SEM at a voltage of 5 kV.

### Evaluation of Optical and Antibacterial Properties

The diffuse reflectance UV-VIS spectra were measured using a UV-VIS spectrophotometer (Cary-300) equipped with an integrating sphere. The scanning range was from 800 to 300 nm.

For bactericidal experiments, the hybrid wood materials were machined into round shape with diameter of 10 mm. The bacterial suspension (*Escherichia coli*) was applied uniformly on the surface of a nutrient agar plate before placing the samples on the plate (1 control and 2 treated samples per plate). The plates were incubated at 37 °C for 24 h, after which the average diameters of the inhibition zone surrounding the samples were measured with a ruler with up to 0.1 mm resolution.

## Results and Discussion

### X-ray Diffraction Analysis

**Fig. 3:**
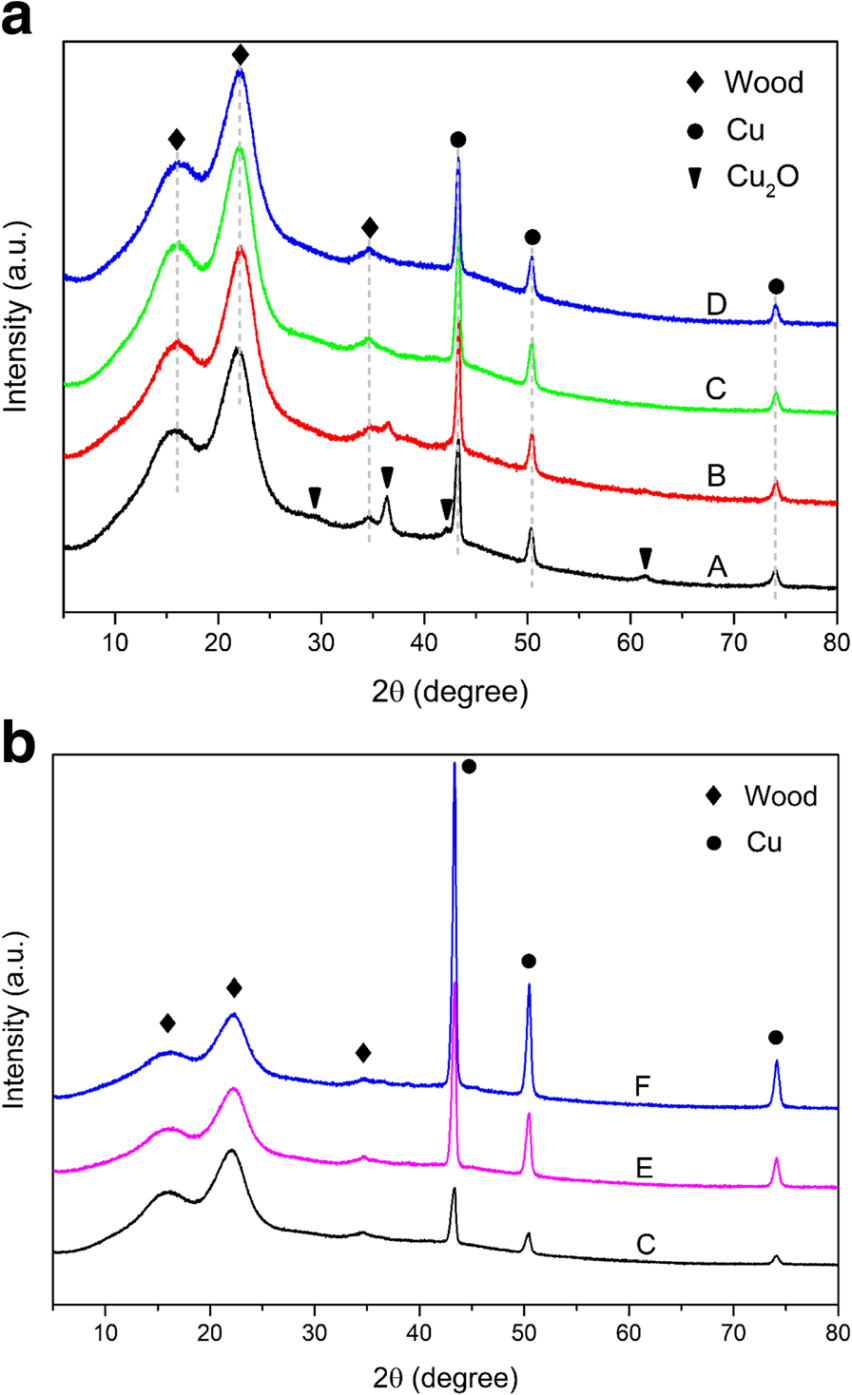
XRD patterns of samples in **a** groups A–D, **b** groups C, E, and F

Figure 3a displays the XRD patterns of samples in groups A, B, C, and D. For all samples, obvious characteristic peaks appearing around 15.9°, 22.1°, and 34.5° were assigned to (101), (002), and (040) planes of cellulose, respectively [27]. The characteristic peaks around 43.3°, 50.4°, and 74.1° were attributed to the (111), (200), and (220) planes of Cu, respectively, which can be indexed to the *fcc* structure of Cu (JCPDS No. 85–1326) [10, 28]. However, some small peaks at around 29.7°, 36.4°, 42.2°, and 61.4° only appeared in samples A and B, associated with the (110), (111), (200), and (220) planes of Cu_2_O NPs, respectively [10]. These phenomena indicated that the products of nanoparticles depended strongly on the initial OH^−^ concentration. At a lower concentration, the products were mainly Cu and Cu_2_O NPs. As OH^−^ concentration increased, Cu_2_O NPs gradually decreased. When the OH^−^ concentration reached 1.0 mol/L or higher, all the Cu_2_O contaminants disappeared and only Cu NPs remained in the products. Generally, the metallic Cu can be synthesized through redox reaction between Cu^2+^ and NaBH_4_ [29]. The presence of OH^−^ in this system is to adjust the pH and accelerate the reaction in water [30]. NaBH_4_ could react with H_2_O when pH is below 9.5, which will weaken its reducing capacity [31]. Therefore, the pH was adjusted to 10–12 by NaOH [5]. In addition, the grain size of Cu NPs will be decreased with the increase in pH value [31]. However, wood components are sensitive to the high alkaline condition and are degraded by NaOH, which will reduce the concentration of OH^−^. Thus, higher concentration of OH^−^ was needed to synthesize pure Cu NPs in wood template. At high OH^−^ concentration, Cu^2+^ was transformed to Cu(OH)_2_ as precursor, then reduced by NaBH_4_, which could be confirmed from the solution color change from deep blue to colorless [32]. However, the transition of metal Cu cannot usually be obtained via the reduction of simple Cu salts without other reagents such as protective polymers with functional groups. Instead, the reduction is more likely to stop at the Cu_2_O stage due to the presence of a large number of water molecules [33]. In this case, the generation of pure Cu NPs may have been due to the wood template: firstly, the hierarchical structure of wood contributed to the assembly of NPs; and secondly, the electron-rich features of phenolic and hydroxyl groups in wood components exerted a reducing and stabilizing effect on Cu NPs [25].

To study the effect of Cu^2+^ concentration on the products, XRD patterns of samples in groups C, E, and F are shown in Fig. 3b. As the Cu^2+^ concentration gradually increased, the intensity of Cu crystal diffraction peaks increased accordingly, indicating that more Cu NPs were generated in the wood template. The crystallite size was calculated using the Scherrer equation,

$$ D=\frac{K\lambda}{\beta \cos \theta } $$

where *D* is the crystallite size, *k* is Scherrer constant (= 0.94 assuming that the particles are spherical), λ is the wavelength of the X-ray radiation (0.15418 nm), β is the full width of the peak at half maximum, and θ is the diffraction angle [10].

The average diameter of Cu NPs was calculated according to the peaks of (110), (200), and (220). The average grain size of Cu NPs in samples C, E, and F were estimated to be approximately 19.5, 19.7, and 21.3 nm, respectively (Table 2). Although the concentration of Cu^2+^ increased significantly, the derived Cu NPs exhibited similar grain size, possibly because the hierarchical structure of wood restricts the growth of Cu NPs [34].

**Table 2:** The grain size of Cu NPs in group C, E, and F

Groups	Grain size (nm)		Significance^a^
	Initial synthesis	After storage	
C	19.54 ± 1.87	18.34 ± 1.59	NS^b^
E	19.74 ± 2.41	18.93 ± 1.18	NS
F	21.36 ± 3.09	20.08 ± 1.76	NS

^a^One-way analysis of variance at α = 0.05

^b^NS means not significant

**Fig. 4:**
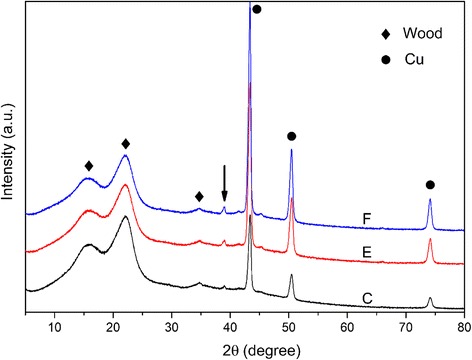
XRD patterns of groups C, E, and F after 1 year stored in ambient conditions

After being stored for 1 year in ambient conditions, the stability and grain sizes of Cu NPs in the wood samples were evaluated. Figure 4 displays the XRD patterns of samples in groups C, E, and F. The main signals of Cu NPs in the wood samples were similar to those shown in Fig. 3; only the small peak appearing at 38.9° could be related to the CuO (see arrow in Fig. 4). From Table 2, the average grain size of Cu NPs in samples C, E, and F were similar to the initial sizes. There was no significant change after storage according to the one-way analysis of variance. These results indicated high stability of Cu NPs in the wood structure. Therefore, the problems of oxidation and aggregation could be circumvented by the use of wood templates, presumably due to the protective effect of the wood’s original structure and components. In addition, the oxidation layer on the wood surface may also contribute to the stability of the internal Cu NPs.

### Morphology Analysis

**Fig. 5:**
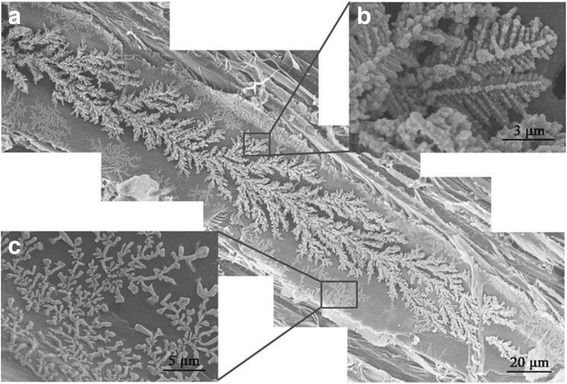
FE-SEM observations of Cu NPs in poplar wood structure (group F). **a** The Cu nanostructures along wood cell lumen. **b**, **c** The magnifications of the Cu nanostructures

**Fig. 6:**
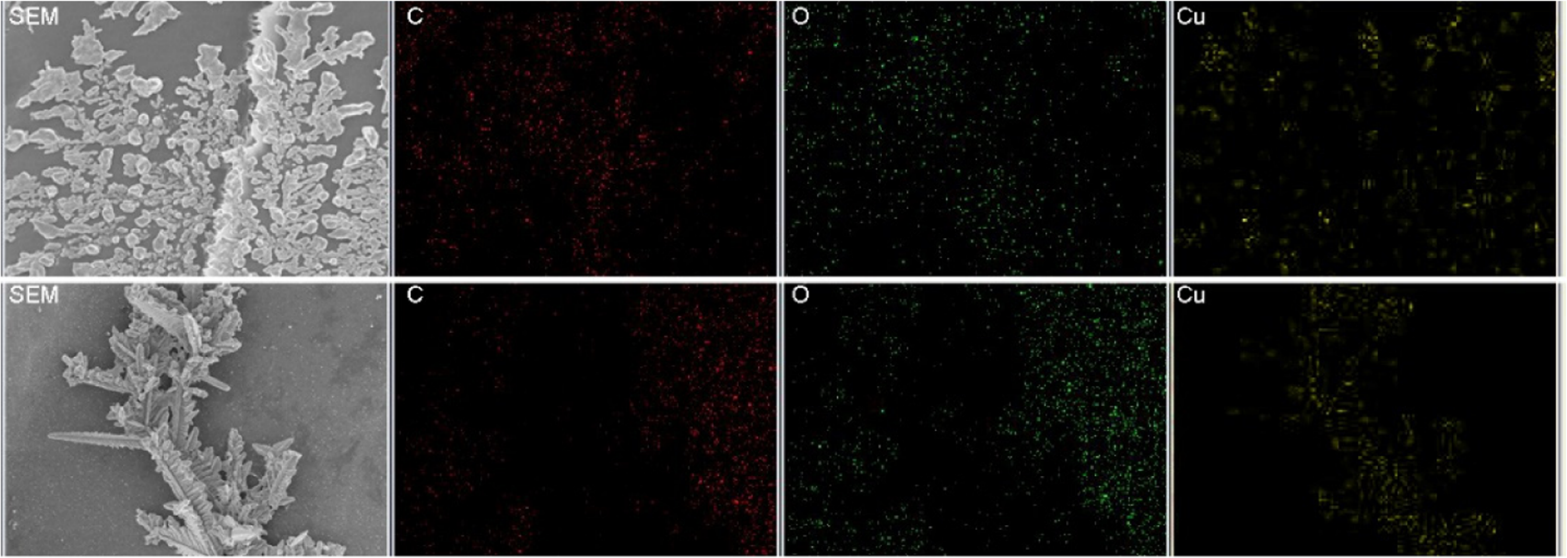
SEM/EDS analysis of Cu distribution in wood structure. The top line is the analysis of the secondary structures on wood lumen wall and the bottom line is the analysis of the main structures on wood lumen wall

The morphology of Cu nanostructures was assessed via FE-SEM; all samples exhibited a similar assembled structure and morphology of Cu nanostructures, as shown in Fig. 5. In Fig. 5a, the agglomerates exhibited a 3D structure along the wood cell lumina that consisted of corncob-like deposits. In addition, many secondary structures adhered to the walls of the cell lumina. Figure 5b, c displays the magnifications of the structures. The pristine wood cell lumen wall was smooth as shown in Fig. 1b. Therefore, the agglomerates on the cell lumen wall could be the assembly of Cu NPs, confirmed by the EDS analysis (Fig. 6). Due to wood’s anisotropic structure, the assembly was oriented, which could explain the anisotropic properties of the materials [23].

**Fig. 7:**
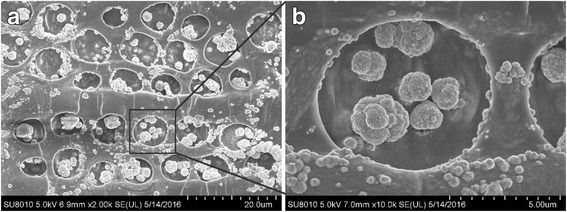
FE-SEM observations of Cu NPs in pits of wood (group F). **a** 2k magnification, **b** 10k magnification

Figure 7 shows the FE-SEM observation of nanostructures in pit of wood. In contrast to that in cell lumen, the Cu NPs in pits were assembled into small spherical agglomerates with diameter from 1 to 2.5 μm. From the magnified image in Fig. 7b, more smaller agglomerates with diameter < 500 nm were adhered to the wall of pit structure. These small and spherical agglomerates could be attributed to the inhibition effect of wood pit structures. It was possible to utilize the wood’s inherent architecture to synthesize the NPs and influence crystallization to some extent, where the NPs no longer resembled the commonly encountered morphologies obtained from classical precipitation reactions in the absence of templates. On the other hand, poplar wood could be endowed with the catalytic, optical, antimicrobial, and electrical conducting properties of Cu NPs’, which would expand wood applications. Previous research has suggested that most Cu NPs are too large to penetrate the wood structure, and their distribution is uneven. Nonetheless, this method could provide a potential approach for fabricating a uniform hybrid wood material via in situ chemosynthesis.

### Optical and Antibacterial Properties

**Fig. 8:**
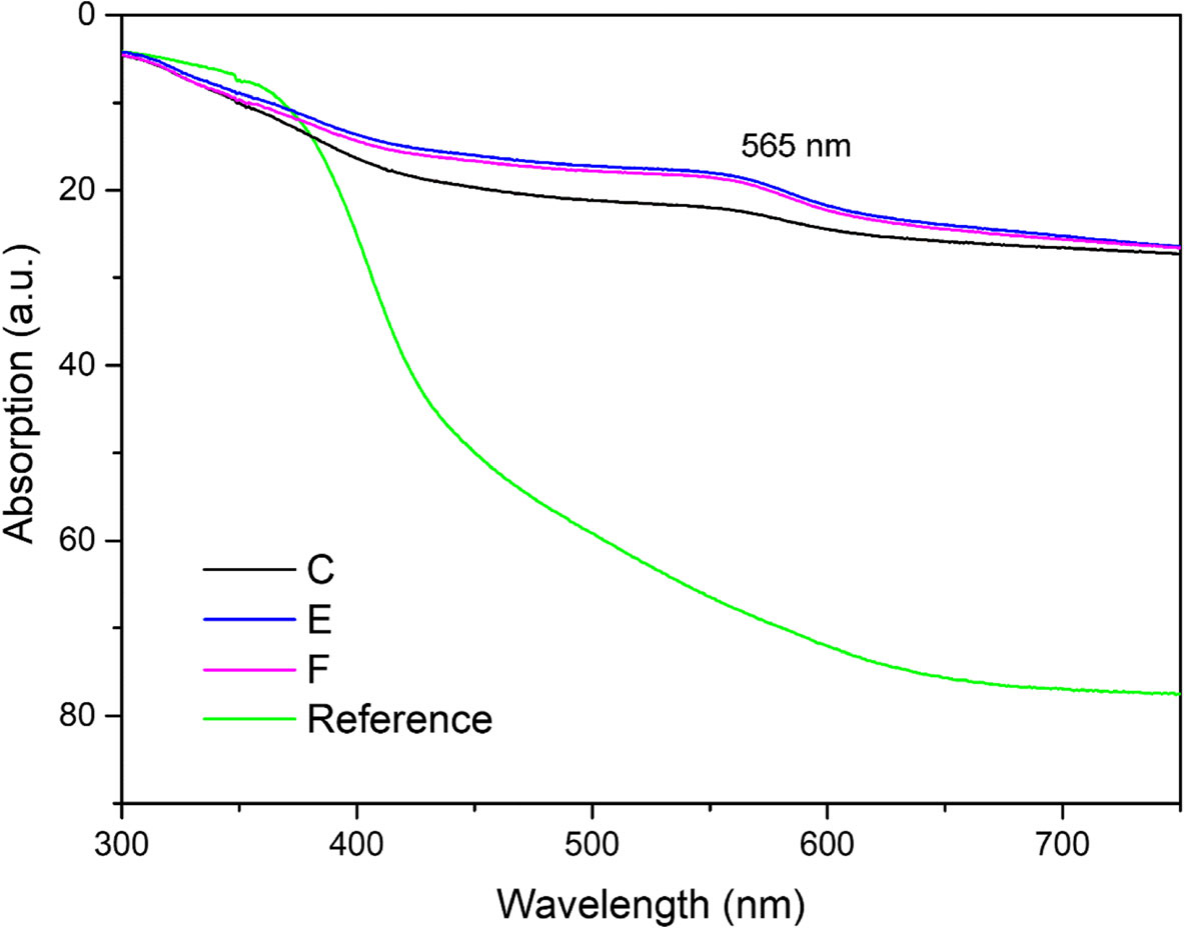
UV-Vis spectra of original wood and hybrid wood materials

**Fig. 9:**
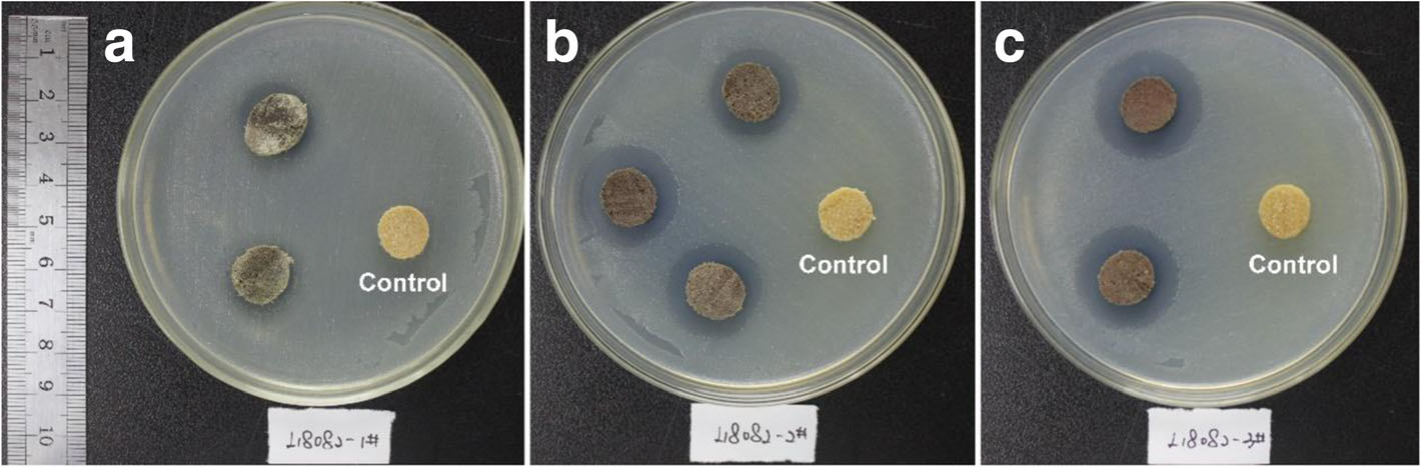
Antibacterial testing of original wood and hybrid wood materials. **a** Group C. **b** Group E. **c** Group F

Figure 8 shows the UV-vis diffuse reflectance curves of the original and hybrid wood materials. The absorption intensity of the hybrid wood materials was higher than that of the control samples. The absorption maxima reached 565 nm for the hybrid wood materials, which was more remarkable in groups E and F due to the higher amount of Cu NPs. This result concurred with the reported plasmon band of dark red Cu NPs in the 560 to 570 nm range [35]. From Fig. 9, in contrast to the control, treated samples in all groups clearly showed zones of inhibition, indicating an antibacterial property against *Escherichia coli*. The average widths of the inhibition zone were 0, 3.2, 4.8, and 6.2 mm for control, groups C, E, and F, respectively. Apparently, the antibacterial property was increased with the increase in the concentration of Cu NPs in wood samples. These results indicated that the incorporation of Cu NPs endowed wood with high antibacterial properties. Thus, the incorporation of Cu NPs can provide wood with optical and antibacterial properties, and other potential properties (e.g., UV resistance as reported in [36]) could also be introduced.

## Conclusions

To leverage the inherent hierarchical, anisotropic architecture, and electron-rich components of wood, pure Cu NPs were derived with unique shapes and sizes through wood template methods. The Cu NPs exhibited a 3D structure along the wood cell lumina that consisted of corncob-like Cu deposits. The nanoparticle products depended strongly on the initial OH^−^ concentration. With an increase in OH^−^ concentration, Cu_2_O gradually decreased and Cu remained. As the Cu^2+^ concentration increased gradually, more Cu NPs were generated in the wood structure. The assembled structure of NPs invariably exhibited corncob-like Cu deposits in the wood templates. Due to the unique structure and components of wood, the oxidation and aggregation of Cu NPs could be circumvented. Additionally, this new hybrid wood material, combined with the advantages of wood and Cu nanostructures, exhibited remarkable optical and antibacterial properties.
